# PReS-FINAL-2106: Clinical psoriatic arthritis of specific children

**DOI:** 10.1186/1546-0096-11-S2-P118

**Published:** 2013-12-05

**Authors:** S Chebisheva, A Meleshkina

**Affiliations:** 1 FMSMU, Moscow, Russian Federation

## Introduction

There were 28 patients with psoriatic arthritis observed amongst the children at the Hospital of the I.M. Sechenov First Moscow State Medical University over the last 10 years.

## Objectives

There were immediate tasks needing to be addressed to reveal the specific clinical processes by which the children with psoriatic arthritis were affected by the disease, by its formation, its skin and joint syndromes, and the variations in the initiation and peaking of the disease.

## Methods

All the children were given general, biochemical, and immunological blood tests. In addition to these, they also received magneto-acoustic tomography (MAET) and X-raying of the affected joints.

## Results

The majority of the children (53%) were found to have acquired the disease at about the age of 6-1/2 years. The median age for the start of the disease was 6.2 +/- 0.5 years, with the youngest being at 4 months and the oldest at 15 years. The mean duration of the disease was 6.7 +/- 0.3 years. In 29% of the cases, the disease began from a skin infection. The effect on the joints began after 2.4 +/- 0.3 years. The joint syndrome was observed in 70.6% of the children with related skin indications showing up on average after 4.5 +/- 0.8 years. Notably, one female patient afflicted with arthritis for 8 years had no signs of skin overpatching.

Our research showed that for 17.6% of the patients the allergoseptic debut of the disease was accompanied by fever, typical rash, and lymphadenopathy. According to published scientific data, the hepatolienal syndrome, or any involvement of the disease with the inner organs, is not typical of psoriatic arthritis. In 11.8% of the patients there was a general deterioration of the joints including the cervical parts of the vertebral column as shown in Still's syndrome type. In 70.6% of the cases, oligoarticular asymmetrical joint syndrome was found to have spread from an initial affection of the ankle joints, to the knee joints, and the proximal interphalangeal joints and hip joints.

Our findings indicate that psoriatic arthritis varies its course in different patients. In 47% of the children, the disease was characterized by high laboratorial and clinical activity with the exacerbation of joint and skin alteration increasing up to 5 to 6 times per year. In 53%, the process was much less pronounced with a positive effect from standard antirheumatic therapy being observed.

At the height of the disease at 3 to 4 years from beginning the observations, 41.2% of the patients were diagnosed with a symmetrical oligoarthritis, spondyloarthritis was reported in 23.5% with affection occurring in the peripheral joints, the ankle joints, knee joints, and the interphalangeal joints, and in 11.8% there was arthritis mutilans.

Our research showed that in 47% of the patients, the disease symptoms corresponded to the 1st stage of the Stein-Broker rentological scale, and in the other 53% to the 3rd and 4th stages. It should be noted in passing, that periarticular osteoporosis is effectively indistinguishable in patients from psoriastic arthritis.

## Conclusion

The course of psoriatic arthritis in children is unpredictable and different from the same disease in adults.

## Disclosure of interest

None declared.

